# Enhancement of pharmacokinetic and pharmacological behavior of ocular dorzolamide after factorial optimization of self-assembled nanostructures

**DOI:** 10.1371/journal.pone.0191415

**Published:** 2018-02-05

**Authors:** Enas A. M. R. Afify, Ibrahim Elsayed, Mary K. Gad, Magdy I. Mohamed, Abd El-Moneim M. R. Afify

**Affiliations:** 1 National Organization for Drug Control and Research (NODCAR), Giza, Egypt; 2 Department of Pharmaceutics and Industrial Pharmacy, Faculty of Pharmacy, Cairo University, Cairo, Egypt; 3 Department of Pharmaceutical Sciences, College of Pharmacy, Gulf Medical University, Ajman, United Arab Emirates; 4 Biochemistry Department, Faculty of Agriculture, Cairo University, Cairo, Egypt; University of South Alabama Mitchell Cancer Institute, UNITED STATES

## Abstract

Dorzolamide hydrochloride is frequently administered for the control of the intra-ocular pressure associated with glaucoma. The aim of this study is to develop and optimize self-assembled nanostructures of dorzolamide hydrochloride and L-α-Phosphatidylcholine to improve the pharmacokinetic parameters and extend the drug pharmacological action. Self-assembled nanostructures were prepared using a modified thin-film hydration technique. The formulae compositions were designed based on response surface statistical design. The prepared self-assembled nanostructures were characterized by testing their drug content, particle size, polydispersity index, zeta potential, partition coefficient, release half-life and extent. The optimized formulae having the highest drug content, zeta potential, partition coefficient, release half-life and extent with the lowest particle size and polydispersity index were subjected to further investigations including investigation of their physicochemical, morphological characteristics, *in vivo* pharmacokinetic and pharmacodynamic profiles. The optimized formulae were prepared at pH 8.7 (F5 and F6) and composed of L-α-Phosphatidylcholine and drug mixed in a ratio of 1:1 and 2:1 w/w, respectively. They showed significantly higher C_max_, ${AUC}_{0}^{24}$ and ${AUC}_{0}^{\infty }$ at the aqueous humor with extended control over the intra-ocular pressure, when compared to the marketed product; Trusopt^®^. The study introduced novel and promising self-assembled formulae able to permeate higher drug amount through the cornea and achieve sustained pharmacological effect at the site of action.

## Introduction

Glaucoma is an ophthalmic disease associated with an increase in the intra-ocular pressure (IOP). Persistent high IOP accompanied with glaucoma can damage the optic nerve with time [1]. Untreated glaucoma may lead to blindness within few years [2]. The anti-glaucoma therapy requires continued administration of medicines for long time and so, sustained release systems are needed to help in keeping constant drug levels at the site of action and enhancing patient compliance through decreasing the dosing frequency [3].

Dorzolamide hydrochloride (DH) is a carbonic anhydrase inhibitor used in treatment of glaucoma through decreasing the production of aqueous humor [4]. DH is 20 times more active than acetazolamide in lowering the IOP when applied topically [5]. The drug is available in the market as eye drops; Trusopt^®^ (Merck, N.J., USA) containing 2% DH in the form of aqueous solution buffered at pH 5.6 [6]. Trusopt^®^ is administered three times a day to achieve a sufficient control over the IOP but this can exaggerate any possible side effect and affect the patient compliance [7]. Reformulation of DH in the form of sustained nanoparticles or nanovesicles may enhance patient compliance, pharmacokinetic and pharmacological behavior [8].

Self-assembly drug nanostructures (SADN) is a well-defined stable structure molecules formed under thermodynamic and kinetic conditions through noncovalent interactions, electrostatic interactions, hydrophobic interactions, hydrogen bonding, -*π* stacking, and Vander Waals forces, etc., to maintain molecules at a stable low-energy state [9]. Recently, many self-assembly drug nanostructures have been synthesized from biomaterials including carbohydrates, nucleic acids, and peptides to achieve a better understanding of the self-assembly mechanism and utilize them for several biomedical applications such as tissue regeneration, drug delivery, and biosensors [10]. Self-assembly drug nanostructures create new materials with suitable morphologies and desired functions through single-molecule design and this results in controlling the bulk properties of the resultant material by modulating individual monomeric building blocks [11]. SADN are considered superior over other encapsulating nanoparticles and/or nanovesicles in having higher drug loading capacity, lower drug leakage and better permeation capabilities through different biological membranes [12].

The aim of this study is to develop SADN of two amphiphilic compounds; DH and L-α-Phosphatidylcholine (PC). The prepared SADN is proposed to have sustained *in vitro* release profile, superior permeation power and extended *in vivo* drug action when compared to the conventional drug solution available in the market.

## Materials and methods

### Materials

Dorzolamide Hydrochloride (DH) was kindly gifted from Jamjoom Pharma, Jeddah, Saudi Arabia. L-α-Phosphatidylcholine (PC) was purchased from Sigma–Aldrich, St. Louis, USA. All other chemicals and solvents were of analytical grade, and utilized without further purification or processing. All rabbits used in the experiment were obtained from Faculty of Agriculture, Cairo University, Cairo, Egypt, and were bred for research purposes.

### Preparation technique

The self-assembled drug nanostructures (SADN) were prepared using a modified thin-film hydration technique [13]. Briefly, DH (200 mg) alone or in a mixture with PC in ratio 1:1 and 1:2 w/w was dissolved in 10 mL dichloromethane as shown in Table A in S1 File. The yielded solution was evaporated under reduced pressure in a rotary evaporator (Rotavapor, Heidolph VV 2000, Burladingen, Germany) equipped with a 50 mL rounded-bottom flask rotating at 100 rpm IN thermostatic water bath (40 ^o^C). The formed residues were hydrated for 1 h using 10 mL of either citrate buffer (pH 5.4), Tris-HCl buffer (pH 7.05) or borate buffer (pH 8.7) to yield a final drug concentration of 2% w/v. The prepared dispersions were sonicated for 1 min, filtered on filter paper to remove any aggregates and kept in refrigerator for characterization.

### Statistical design of the study

Response surface design was utilized to investigate the influence of formulation variables on physicochemical characteristics of the prepared SADN formulae using Design-Expert^®^ 7 Software (Stat-Ease Inc., Minneapolis, MN, USA). As displayed in Table 1, the effects of pH (X_1_) and drug to polymer ratio (P/D: X_2_) on the drug content (DC: Y_1_), particle size (PS: Y_2_), polydispersity index (PDI: Y_3_), zeta potential (ZP: Y_4_), partition coefficient (K: Y_5_), release half-life (T_50%_: Y_6_) and release extent (RE: Y_7_). Based on that, the composition of 6 formulae were obtained to be prepared and characterized. Consequently, the selected composition(s) was/were determined based on the desirability function for simultaneous optimization of the traced responses.

**Table 1:** Composition of the prepared SADN formulae based on the response surface design and the measured responses.

Formulae	Factors			Responses					
	pH	P/D ratio	DC (%)	PS (nm)	PDI	ZP (mV)	K	T_50%_ (min)	RE (%)
F1	Low	Low	69.22	604.1	0.575	-39.3	0.188	605	88.34
			61.10	594.8	0.603	-41.7	0.179	627	84.61
F2	Low	High	83.63	385.8	0.273	-40.4	0.125	840	76.26
			81.09	372.9	0.312	-41.5	0.121	859	75.81
F3	Medium	Low	23.97	386.3	0.499	-51.3	1.914	1332	78.32
F4	Medium	High	30.77	190.4	0.475	-46.0	2.821	1569	68.20
F5	High	Low	92.64	237.4	0.256	-55.5	0.852	243	82.84
			97.81	236.8	0.240	-60.7	0.864	274	86.67
F6	High	High	98.28	103.9	0.714	-54.5	2.330	355	85.28
			99.33	100.1	0.670	-57.8	2.180	316	81.74

### *In vitro* characterization of the prepared self-assembled drug nanostructures

#### Drug content

Samples were taken from each prepared formula and diluted with methanol (100 times). The diluted dispersions were kept overnight in a thermostatic shaking water bath (Gesellschaft für Labortechnik, Burgwedel, Germany) running at a speed of 100 rpm [14]. Then, clear aliquots were taken after filtration using 100 nm membrane filters (Büchi Labortechnik AG, Flawil, Switzerland). The clear solutions were analyzed for its drug content using UV spectrophotometer (SPD-10 A, Shimadzu, Tokyo, Japan) at λ_max_ of 254 nm. Drug content of each formula was calculated using the following equation:

$$Drug\;content\;\%=\frac{A_{PS}}{A_{TS}}x\;100$$ (1)

where, A_PS_ and A_TS_ are the practical and theoretical drug amount in the employed sample volume, respectively.

#### Particle size, particle size distribution and zeta potential

Particle size, particle size distribution and zeta potential of the optimized formulae were measured using dynamic light scattering technique (Nano ZS-90 Zetasizer, Malvern Instruments, Malvern, UK) after dilution of each nanodispersion with bi-distilled water in ratio 1:10 v/v.

#### Partition coefficient

Effects of the pH and D/P ratio on the drug partition coefficient were determined based on shake-flask technique [15]. Samples of the prepared formulae were shaken with equal volumes of n-octanol for 24 h in a thermostatic shaking water bath. The mixtures were shaken at 100 rpm and temperature 32 ^o^C to simulate the surface temperature of the eye. Then, the aqueous phase was separated and the drug concentration was determined using UV spectrophotometer at the predetermined drug λ_max_. The partition coefficient (K_o/w_) was calculated based on the following equation:

$$K_{o/w}=\frac{C_{oct.}}{C_{wf}}=\frac{C_{wi}-C_{wf}}{C_{wf}}$$ (2)

where, C_oct._ and C_wf_ are the drug concentrations in octanol and water at equilibrium, respectively, while, C_wi_ is the initial drug concentration in water. The percent of solubility in both layer shown in Table B in S1 File.

#### *In vitro* release

The drug release rate and extent from each formula were studied and compared to the equivalent drug solution using modified Franz diffusion cell. Capacity of the acceptor cell was 10 mL with a 0.5 cm aperture facing the donor cell. Overnight soaked dialysis tubing cellulose membrane (Typical molecular weight cut-off = 14,000 Da, Sigma–Aldrich, St. Louis, USA) were fixed between the two cell after filling of the acceptor one with simulated tear fluid (pH 7.4) rotating at speed 100 rpm using Teflon coated magnetic bead [16]. The temperature of the water jacket surrounding the acceptor cell was adjusted at 32 ± 0.5°C. Samples (0.2 mL, each) were taken at different time intervals (15, 30 min., 1, 2, 3, 4, 5, 6, 7, 8 and 24 h), filtered, diluted and then spectrophotometrically analyzed the predetermined DH λ_max_. Each formula was tested thrice and the average DH release percentages were calculated and plotted against time to demonstrate the drug release profiles from different formulae.

The release kinetics were determined based on substitution in the different order equations (zero, 1^st^ and Higuchi diffusion) to compare the correlation coefficient and determine the most representative order [17]. Based on the selected order, the release half-life (T_50%_) was calculated and utilized to compare the release rate of DH from different formulae.

### *In vitro* characterization of the optimized SADN formulae

#### Differential scanning calorimetry and X-ray diffraction

Samples were taken from DH, PC and the optimized formulae; and placed in sealed aluminum pans. Temperature was raised up to 300°C under a cover of nitrogen gas with heating rate of 10°C/min using differential scanning calorimeter (Mettler-Toledo International Inc., OH, USA). The samples thermograms were constructed and the DH peak(s) was/were traced in each thermogram for presence of any shift or disappearance.

Patterns of X-ray diffraction were determined using MD-10 mini-diffractometer (MTI Corporation, CA, USA) operating at a voltage 25 kV and a current of 30 mA [18]. The test was performed for DH, PC and the optimized formulae.

#### Transmission electron microscopy

The surfaces of the optimized nano-complexes were visualized using transmission electron microscope (JEM-2100, JEOL, Tokyo, Japan) operated at a voltage of 100 kV. This was done after spreading the taken samples over a copper grid, staining them with 1% phosphotungistic acid and air-drying of the stained samples.

### *In vivo* characterization of the optimized SADN formulae

#### Animals

The animal experiments were done in full compliance with regulatory principles and after approval of ethics committee of Faculty Pharmacy, Cairo University (Approval No. PI 1181). Male New Zealand albino rabbits weighting 2–3 kg were housed at controlled temperature (25 ± 2°C), and humidity (60 ± 5%), with a 12/12 h light-dark cycles. Nine animals were employed in the pharmacological study and six in the bioavailability evaluation.

#### Bioavailability enhancement

One drop (50 μl) from each optimized formula was applied to right eye of the employed animals after being randomly divided into two groups, one group per formula. Trusopt^®^ eye drop was applied to the left eyes of all animals as a reference to act as a reference and evaluate the improvement of the DH pharmacokinetics absorbed from the optimized formulae. The rabbits’ eyelids were kindly kept closed for few seconds to prevent dose drainage. After anesthetizing the animals using intramuscularly injected ketamine hydrochloride (50 mg/kg) and xylazine (10 mg/kg), samples of the aqueous humor (50 μl) were withdrawn at the following time intervals: 1, 2, 4, 6, 8, 24 h, using insulin syringe equipped with 29 gauge [19]. Samples were frozen at -20°C till the time of analysis.

Before analysis, the aqueous humor samples containing DH were mixed with the internal standard solution (Mepivacaine hydrochloride, 50 μg/mL) in ratio 5:1 v/v, and then extracted using acetonitrile (HPLC grade) [19]. The organic phase was separated, dried under vacuum using vacuum concentrator (Eppendorf 5301; Hamburg, Germany) and reconstituted by a mobile phase composed of acetonitrile: potassium dihydrogen phosphate (30 mMol) containing 0.1% triethanolamine, pH 3.5, mixed in ratio 20:80 v/v as shown in Figure A in S1 File. Samples were injected at a flow rate of 1 mL/min into HPLC (Shimadzu, Kyoto, Japan) equipped with UV-Visible detector, Hypersil C18 column and 20 μL injector.

DH concentration at each time interval was calculated based on a pre-established calibration curve. The obtained data were subjected to non-compartmental pharmacokinetics analysis using PK solver add-in program [20]. For each curve, the maximum plasma concentration (C_max_), its equivalent time (T_max_), areas under the curves (${AUC}_{0}^{24}$ and ${AUC}_{0}^{\infty }$) and mean residence time (MRT) were calculated.

#### Pharmacological reinforcement

Animals were randomized in three groups, each composed of three rabbits. Experimental glaucoma was induced by four daily intraocular injections of 2% w/v methylcellulose [21]. A single dose (50 μl) of the optimized formulae was applied to the cornea of the first two groups. In addition, equal volume of Trusopt^®^ eye drop was administered to the third group as a reference for the comparison purposes. The intraocular pressure (IOP) of the rabbits were measured using an indentation tonometer (Rudolf Riester GmbH Co., Jungingen, Germany) at different time intervals (0.5, 1, 2, 3, 4, 5, 6, 7, 8 and 24 h) [22]. The normalized IOP pressure was calculated and the obtained data were recorded as the mean values ± standard deviations.

#### *In vitro*–*in vivo* correlation (IVIVC)

Multiple levels C correlation was adopted to correlate the percentages of the *in vitro* release at each time interval with each of the area under the curve of the pharmacokinetic study and the normalized IOP measured during the pharmacological study [23]. Data were plotted and the most fitting regression model will be considered to determine the correlation coefficients.

## Results and discussion

### Formulation and preparation of SADN formulae

**Fig 1:**
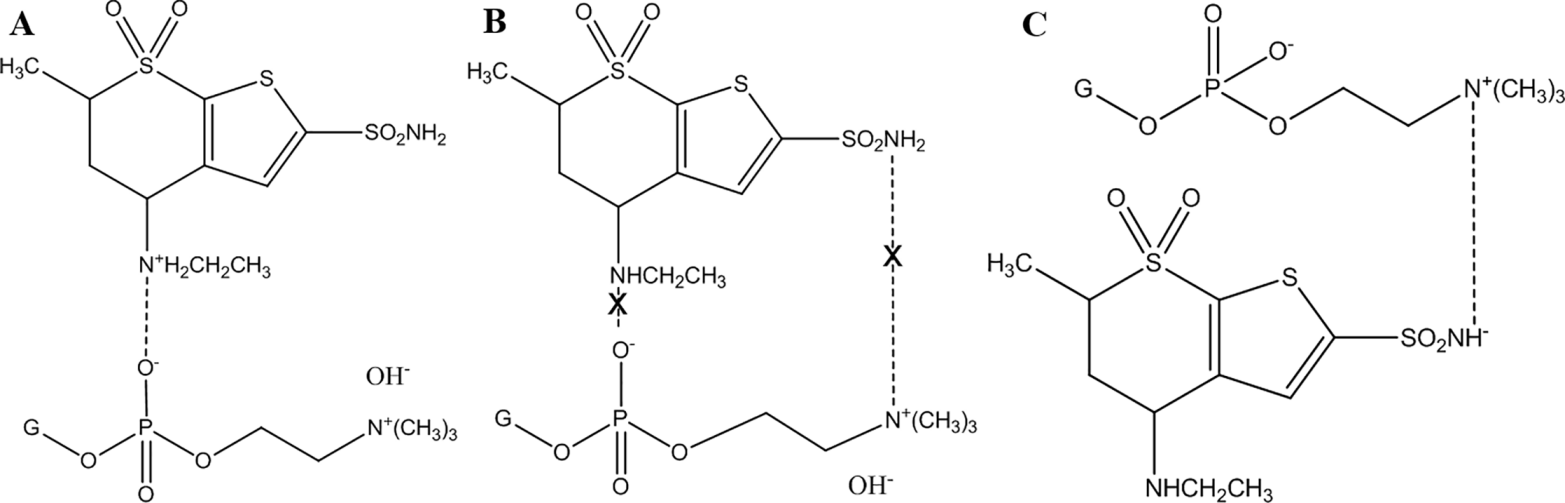
The electrostatic interactions between the drug and phosphatidylcholine at low (A), medium (B) and high (C) pH values.

pH values were selected carefully to provide suitable molecular environment for electrostatic interaction between DH and PC, as illustrated in Fig 1. The drug has two isoelectric points at 6.4 and 8.5 [24]. At pH 5.4, DH carried positive charge on the quaternary ammonium group and had a negative charge loaded on the sulfonamide nitrogen at pH at 8.7 while it is neutral at the intermediate pH (7.05) [25]. On the other hand, PC remained negatively charged nevertheless the surrounding pH because all selected pH values were higher than the isoelectric point of PC (4) [26]. Interaction between the cationic DH and the anionic PC could happen at pH 5.4 while the chance of electrostatic migration was expected to be minimal at pH 7.04 due to the absence of any charge at the drug structure. On the other hand, the negatively charged drug at pH 8.7 was proposed to replace the hydroxyl groups neutralizing the positively charged quaternary ammonium group of PC [27].

### *In vitro* characterization of the prepared self-assembled drug nanostructures

#### Drug content

High drug content was desired to reduce the volume, equivalent to the drug dose, instilled in each eye [28]. The determined values were ranged between 23.97% and 99.33% throughout the prepared formulae. Quadratic model was utilized to analyze the data and validated based on its non-significant lack of fit (*p*-value = 0.5561), high adequate precision (32.29) and the reasonable agreement between the adjusted and predicted r^2^ (0.9854 and 0.9691, respectively) [29]. Results analysis yielded the following polynomial equation:

$$DC=27.37+11.62\;X_{1}+4.83\;X_{2}-3.40\;X_{1}X_{2}+58.02$$ (3)

**Fig 2:**
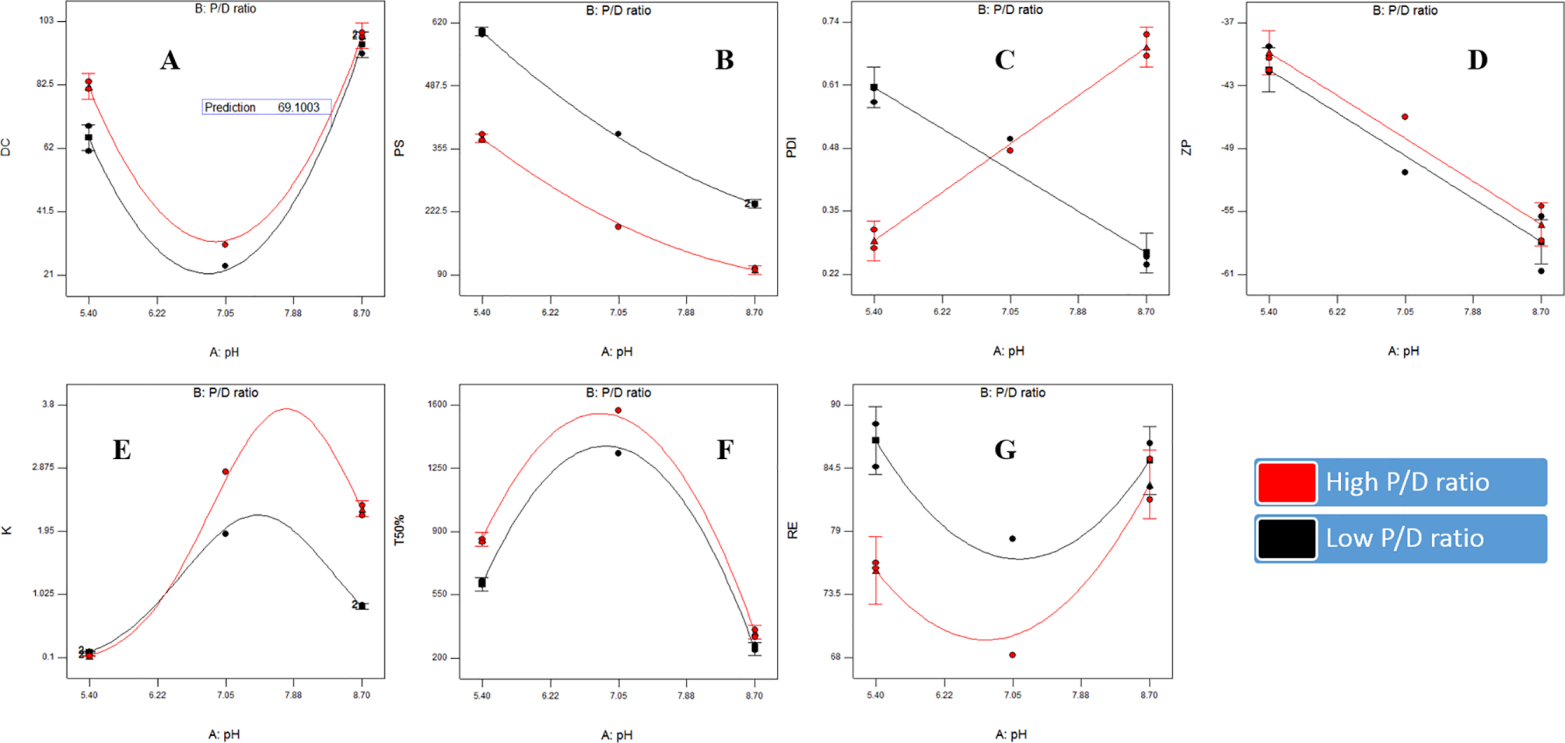
Response surface plots for the effects of pH and P/D ratio on the drug content (A), particle size (B), polydispersity index (C), zeta potential (D), partition coefficient (E), release T_50%_ (F) and release extent (G) of Dorzolamide self-assembled nanostructures.

Factorial analysis of variance showed the presence of significant effects of pH and P/D ratio on the drug content (*p*-values = 0.0002 and 0.0058, respectively). The lowest drug content values were at the medium pH (7.05), as shown in Table 1 and Fig 2A. This could be explained in terms of the low drug solubility at neutral media as per the study performed by Sigurdsson *et al* [25]. The low solubility might lead to the presence of excess undissolved drug in the formula which could be removed by filtration after preparation. On the other hand, increasing the PC ratio lead to a significant increase in the drug content. This could indicate the efficiency of 2:1 w/w P/D ratio (nearer to 1:1 Mol/Mol) to form SADN with the drug when compared to the lower ratio. This could support the equimolar interaction between PC and the candidate drugs as previously stated in several researches [13, 14 and 30].

#### Particle size, particle size distribution and zeta potential

Particle size was preserved in the nano range where values varied between 100.1 and 604.1 nm throughout the prepared SADN formulae, as demonstrated in Table 1 and Fig 2B. Quadratic model was the most fitting one for the analysis of the obtained data with non-significant lack of fit (*p*-value = 0.1162), high signal to noise ratio (adequate precision = 96.87) and good matching between the adjusted and predicted R^2^ values (less than 0.2) [31]. The following equation represents the effect of the traced factors on the particle size:

$$PS=288.35-159.35X_{1}-90.64X_{2}+21.24\;X_{1}X_{2}+41.13X_{1}^{2}$$ (4)

Significant decrease in the particle size was observed with increasing pH and P/D ratio (*p*-value < 0.0001) with significant interaction between the two factors (*p*-value = 0.0004). The correlation between pH and interfacial tension of PC was previously studied by Petelska *et al* [26] where the interfacial tension was maximal at the PC isoelectric point (≈ 4) and gradually declined by increasing pH [26]. Reducing the interfacial tension could facilitate the formation of smaller nanostructures [32]. On the other hand, the decrease in the particle size with increasing P/D ratio might be referred to the previously discussed efficient complexation between the drug and PC at ratio 2:1 w/w which could lead to formation of nanostructures with higher yield and smaller size. This theory could be opposed by the direct relation between particle size and polymer concentration observed in case of conventional nanoparticles, physically encapsulating the drug [33].

Polydispersity index values were extended from 0.240 and 0.714. It was taken as a parameter for evaluating the degree of particle size variation. Data were analyzed using two-factor interaction model. Adjusted and predicted r^2^ were close to each other (0.9604 and 0.9388, respectively) and adequate precision was higher than 4 (18.77) indicating the ability of the selected model to navigate the design space with efficient predictive capability [34]. The representing equation was as follows:

$$PDI=0.46+0.01X_{1}+0.03\;X_{2}+0.03X_{2}+0.18\;X_{1}X_{2}$$ (5)

pH and P/D ratio have no significant effects on polydispersity index as *p*-values were 0.2913 and 0.0535, respectively, although there was an interaction between the two factors (*p*-value < 0.0001), as displayed in Table 1 and Fig 2C. Elevation of pH reduced polydispersity index at P/D ratio 1:1 w/w while polydispersity index were raised with increasing pH at P/D ratio 2:1 w/w, but generally, changes were insignificant.

Zeta potential values lied between -39.3 and -60.7, as illustrated in Table 1 and Fig 2D imparting high physical stability on the nanostructures surfaces through hindering their aggregation during storage [35]. Linear model was the most suitable for the factorial analysis of zeta potential values with non-significant lack of fit (*p*-value = 0.6135) when compared to the pure error indicating that the model fits well [36]. Moreover, r^2^ (adjusted: 0.9250, predicted: 0.8793) and adequate precision (15.02) values confirmed the validity and suitability of the linear model to analyze the zeta potential data. The outputted equation was as follows:

$$ZP=-48.87-8.2X_{1}+0.83\;X_{2}$$ (6)

pH had a significant effect on zeta potential (p-value < 0.0001) while P/D ratio had no significant effect (p-value = 0.2708). Zeta potential increased by elevating pH from 5.4 to 8.7 passing through pH 7.05. This could be explained in the light of the electrostatic interactions occurred at each pH value. The positive drug moieties at pH 5.4 could neutralize the negative charges on PC surfaces minimizing the zeta potential while the negatively charged drug molecules formed at pH 8.7 could add a negative potential on the double bilayer of PC raising the zeta potential up to -60 [27].

#### Partition coefficient

Partition coefficient values ranged between 0.121 and 2.821, as shown in Table 1 and Fig 2E, and statistically analyzed using response surface quadratic model. The model fitting and precision were confirmed through determining the lack of fit significance and adequate precision (*p*-value = 0.1051 and 105.97, respectively). The output quadratic equation was as follows:

$$K=0.36+0.48\;X_{1}+0.07\;X_{2}+0.15\;X_{1\;}X_{2}-0.70\;X_{1}^{2}$$ (7)

Both pH and P/D ratio had significant effect on the partition coefficient values (p-value < 0.0001). The maximum partition coefficients were observed at the neutral pH while the lowest at pH 5.4 with intermediate values at pH 8.7. The relation between partition coefficient and corneal permeability was previously studied in many researches and it was found that the optimum corneal permeation was at partition coefficient range of 2.5–3 [37]. Moreover, the maximum corneal/conjunctival permeability ratio was observed at partition coefficient higher than two [38]. As the maximum measured value in our study was 2.821 which could be optimum for the corneal permeation, the optimization target was set to maximize the partition coefficient.

#### *In vitro* release

**Fig 3:**
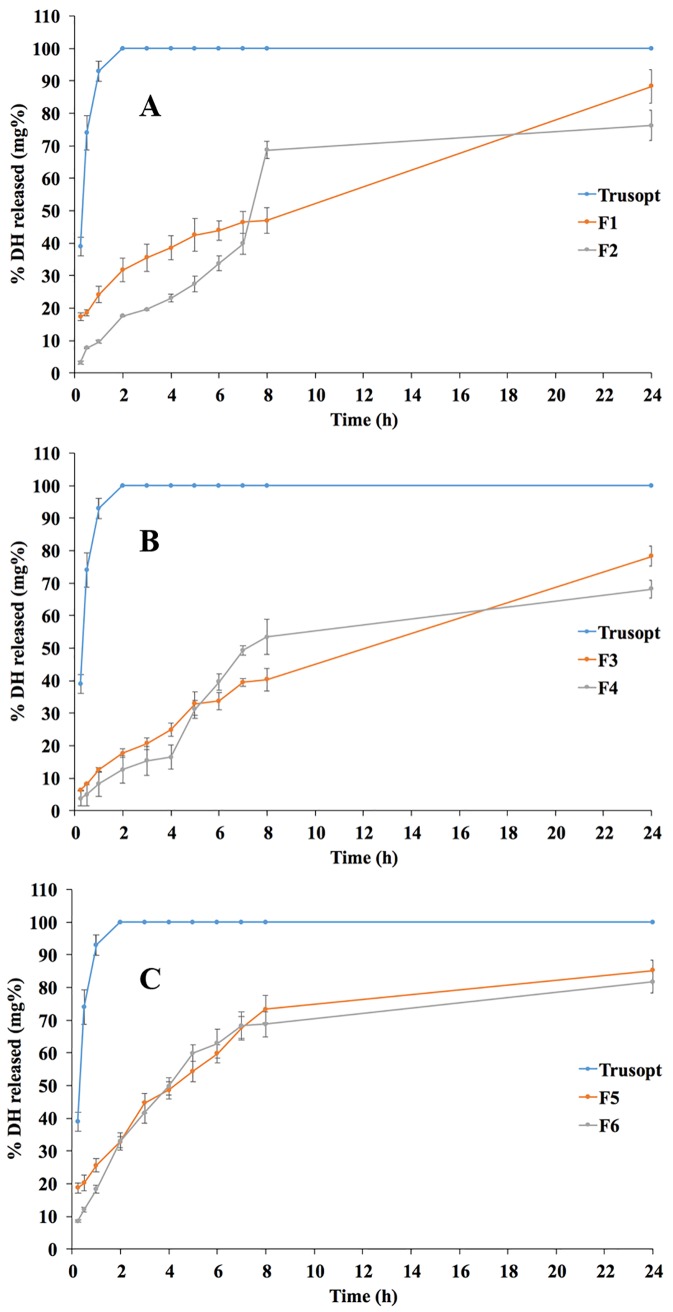
Release profiles of the drug from the formulae prepared at low (A), medium (B) and high (C) pH values.

*In vitro* release through dialysis membranes was taken as a predictive tool for the *in vivo* behavior after instillation into eyes. Two optimization targets were set for the *in vitro* release testing; maximizing the release T_50%_ and extent. At each pH value, the SADN formulae showed slower release rates than the market product, as shown in Fig 3. Furthermore, the release extent values were minimum at the neutral pH. Release T_50%_ values ranged between 243 and 1569 min while release extent values lied between 68.20% and 88.34%, as demonstrated in Table 1, Fig 2F and 2G. To statistically evaluate the data, quadratic model was selected for both responses; T_50%_ and release extent. The selected model was authenticated for the its capability to fit and navigate the design space through ensuring that the lack of fit was non-significantly different from the pure error (p-values = 0.0643 and 0.2997 for T_50%_ and release extent, respectively), in addition to adequate precision exceeding 4 in both responses (61.75 and 10.16 for T_50%_ and release extent, respectively). Factorial equations for the two responses were as follows:

$$T_{50\%}=1450.50-217.87\;X_{1}+85.80\;X_{2}-39.12X_{1}X_{2}-935.62X_{1}^{2}$$ (8)

$$RE=73.6+1.44\;X_{1}-3.35\;X_{2}+2.30\;X_{1}X_{2}+9.43\;X_{1}^{2}$$ (9)

Changing pH had significant influence on the T_50%_ (*p*-value < 0.0001) where the lowest release rate was observed at the neutral pH. This could be correlated to the high partition coefficient and hence, lipophilicity of DH at these conditions that might hinder the drug release to the aqueous medium [39]. On the other hand, both release rate and extent were significantly affected by the P/D ratio (*p*-values = 0.0003 and 0.0065, respectively). This might be attributed to the lipophilic nature of PC and its ability to efficiently associate drug molecules at ratio 2:1 w/w, as discussed before within the drug content data [40].

### Selection of the optimized SADN formulae

Desirability was considered as a statistical tool to select the optimum level for each factor that could simultaneously achieve the desired responses [41]. Optimization process was built on maximizing drug content, zeta potential, partition coefficient, release T_50%_ and extent, in addition to minimizing particle size and polydispersity index. The formulae prepared at pH 8.7 (F5 and F6) had the highest desirability values (0.64 and 0.55, respectively). Consequently, both formulae were selected for following further investigation.

### *In vitro* characterization of the optimized SADN formulae

#### Differential scanning calorimetry and x-ray diffraction

**Fig 4:**
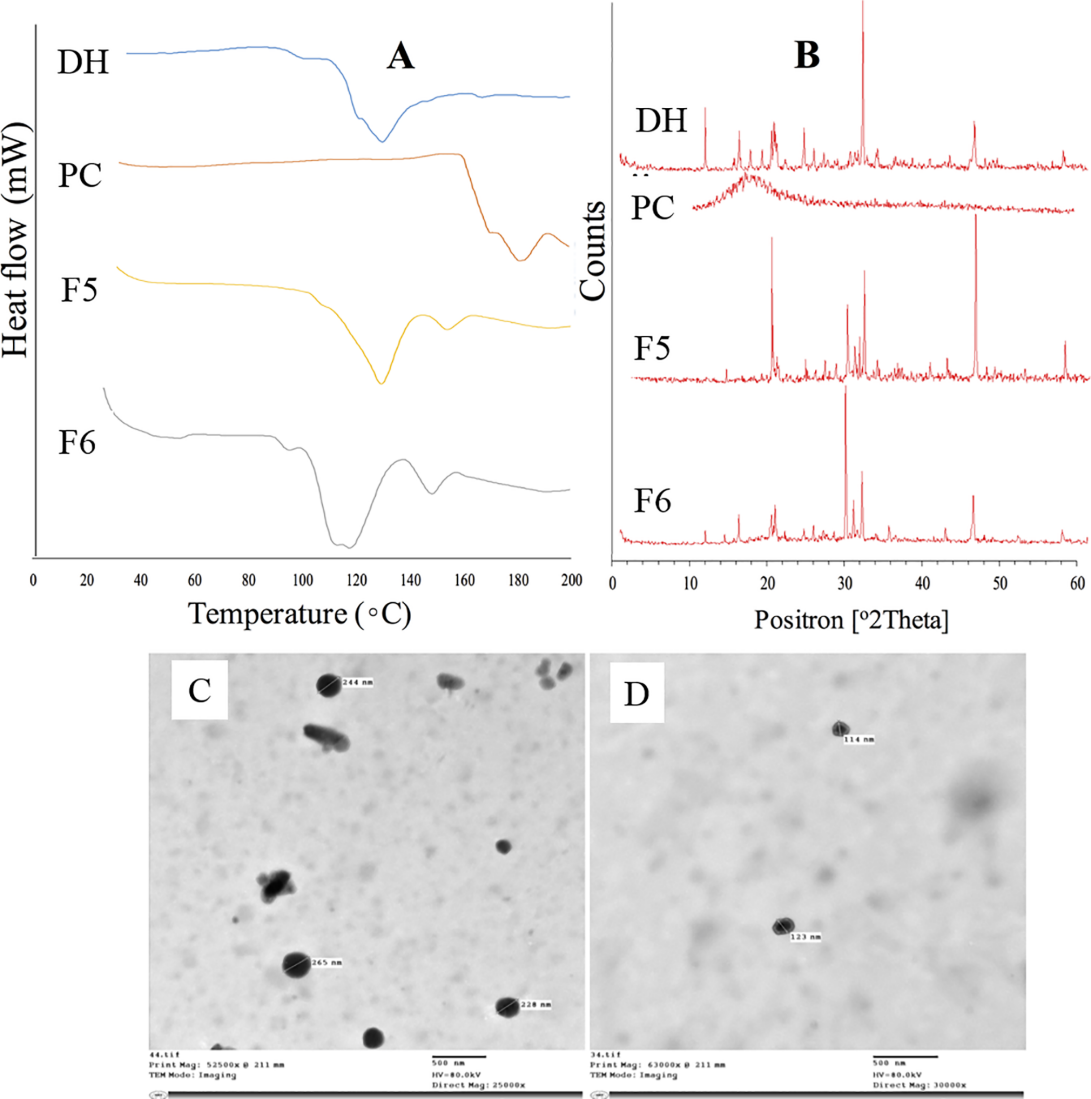
*In vitro* characterization of the optimized formulae using DSC (A), X-ray diffraction (B) and TEM (C and D).

The drug had a characteristic endothermic peak at 131.25 ^o^C, as demonstrated in Fig 4A, while PC had a higher endothermic peak appeared at 181.54 ^o^C. The optimized formulae (F5 and F6) showed down-shifting of the drug and PC peaks. These findings indicated the presence of interaction between DH and PC that could be physical of chemical [42, 43]. X-ray diffraction was performed to investigate nature of the detected interaction and the obtained diffractograms were displayed in Fig 4B. The drug had characteristics peaks at 12.7, 16.9, 20.8, 24.8, 32.1 and 45.8 (2θ), while PC lacked the presence of any peak throughout its diffractogram. These data could point out the crystalline nature of DH and the amorphous nature of PC. In case of F5 formula, the peak at 16.9 (2θ) disappeared while a new peak aroused at 30.0 (2θ). Moreover, two peaks appeared within F6 diffractogram at 21.3 and 30.0 (2θ). Changes in the drug characteristic peaks, especially appearance of new peaks, despite the amorphous PC nature, could elucidate the chemical interaction between DH and PC rather than the drug physical inclusion within the conventional PC liposomes [44, 42].

#### Transmission electron microscopy

Imaging by transmission electron microscope was employed to investigate the morphological characteristics of the optimized SADN formulae. Fig 4C and 4D showed the existence of uniform nanoparticles with a size range matching the data obtained by the Zetasizer analysis. Moreover, almost all particles had spherical outline with smooth surfaces.

### *In vivo* characterization of the optimized SADN formulae

#### Bioavailability enhancement

The pharmacokinetic behavior of the optimized formulae was compared to the market product (Trusopt^®^) to determine the degree of enhancement in the rate and/or extent of drug permeation to the aqueous humor as shown in Table C in S1 File. The optimized formulae did not show sustained *in vivo* profiles relative to the market product as the in terms of the T_max_ and MRT values, as displayed in Table 2. On the other hand, Formulae F5 and F6 had significantly higher C_max_, ${AUC}_{0}^{24}$ and ${AUC}_{0}^{\infty }$ than that of Trusopt^®^ (p-value < 0.001) indicating the capability of the formulated SADN to enhance the corneal permeation. This could be referred to the relatively high partition coefficient of the formed nanostructure that facilitate the transcellular permeation of the complexed drug when compared to the polar drug aqueous solution represented by the market product [45, 46].

**Table 2:** Pharmacokinetic parameters of the optimized SADN formulae compared to the marketed product; Trusopt^®^.

Pharmacokinetic Parameters	Trusopt^®^	F5	F6
T_max_ (h)	6.00 ± 0.0	2.00 ± 0.0	6.00 ± 0.0
C_max_ (μg/ml)	2.70 ± 0.1	4.92 ± 0.3	3.61 ± 0.2
${AUC}_{0}^{24}$ (μg/ml.h)	19.56 ± 0.6	38.21 ± 2.9	53.69 ± 1.3
${AUC}_{0}^{\infty }$ (μg/ml.h)	26.29 ± 1.7	57.00 ± 3.0	71.73 ± 4.5
MRT (h)	17.15 ± 1.1	19.85 ± 1.4	17.06 ± 0.9

#### Pharmacological reinforcement

**Fig 5:**
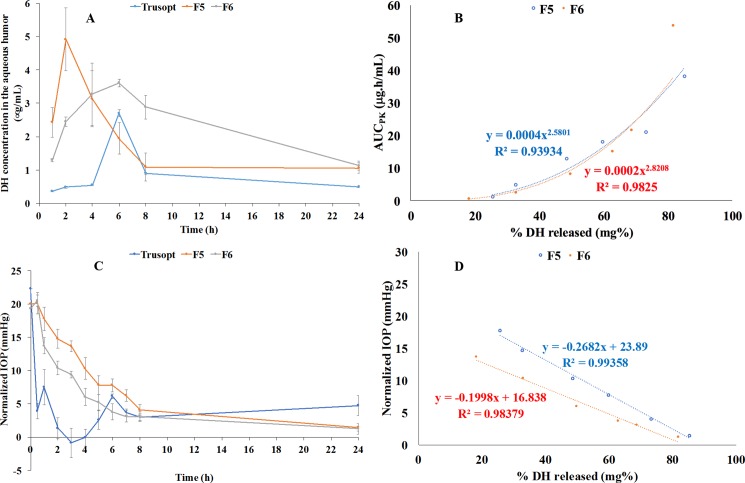
Pharmacokinetic and pharmacological behavior of the optimized formulae (A and C) and their correlation to the *in vitro* release profiles (B and D).

Glaucoma was initiated before applying the different treatments to render the results more realistic and sensitive than working on normal IOP [21]. Trusopt^®^ eye drop rapidly exerted its action and reduced the IOP to its normal value within 2–3 h, as shown in Fig 5C. This was followed by two-phase increase in the IOP where the 1^st^ one was fast and the second represented a gradual rise starting from the eighth hour and up to end of the experiment (24 h). Similar profile was observed by Dinslage *et al* who also studied the effect of Trusopt^®^ (2%) on IOP after ophthalmic application in rabbits’ eyes [47]. On the other hand, the optimized formulae showed a slow decline in the IOP throughout the experiment time (24 h) proving their extended action although their pharmacokinetic profiles were not sustained. The formula F6 (P/D ratio: 2:1) showed better control over the IOP when compared to F5. This could indicate that rapid permeation of the nano-complexed drug was followed by a sustained dissociation of the drug at the receptor site.

#### *In vitro*—*in vivo* correlation (IVIVC)

IVIVC is originally established and performed for orally administered class I drugs having high solubility and permeability [48]. Development of IVIVC for other classes of drug administered by other routes of administration could be breakthrough achievement in the drug development process. In our study, the power regression was found to be the most fitting model to correlate percentages of the *in vitro* release to the area under the curve of the aqueous humor level–time curve with reasonable correlation coefficients of 0.9393 and 0.9825 in case of F5 and F6, respectively, as illustrated in Fig 5B. On the other hand, linear correlation was the most valid model for correlation of the *in vitro* data to the normalized IOP in the optimized formulae with significantly high R^2^ (> 0.98), as demonstrated in Fig 5D. This could imply the high possibility of getting a good correlation between the *in vitro* and *in vivo* data and this can be beneficially utilized during development of new ocular DH nano-systems in the future.

In the present study Dorzolamide as self-assembled drug nanostructures were prepared by a simple and reproducible method. pH values were selected carefully to provide suitable molecular environment for electrostatic interactions between DH and PC. Optimization process was built on maximizing drug content, zeta potential, partition coefficient, release T_50%_ and extent, in addition to minimizing particle size and polydispersity index of the prepared SADN formulae. The formulae prepared at pH 8.7 (F5 and F6) had the higher desirability values and high significant ${AUC}_{0}^{24}$ and ${AUC}_{0}^{\infty }$ than that of Trusopt indicating the capability of the formulated SADN to enhance the corneal permeation. In addition, the optimized formulae exerted sustained lowering effect of the IOP when compared to the marketed product. Finally, the developed formulations are expected to improve the patient compliance, form better dosage regimen and provide optimum maintenance therapy to glaucomatous patients.

## Supporting information

S1 File (Figure A) Aqueous humor sample step preparation for Hplc analysis. (Figure B) Graphical Abstract. (Table A) Ratio of phosphatidylcholine with Dorzolamide Hcl at low (A), medium (B) and high (C) pH values respectively. (Table B) Solubility Studies (Water/Octanol Solubility) of different SADN. (Table C) % Cumulative Release of Trusopt, SADN "F5 & F6".
(DOCX)
