# Supplementary material for: Enhancement of pharmacokinetic and pharmacological behavior of ocular dorzolamide after factorial optimization of self-assembled nanostructures
Source: PLoS One. 2018 Feb 5;13(2):e0191415. doi: 10.1371/journal.pone.0191415 (PMC5798776; doi:10.1371/journal.pone.0191415)
Supplement: S1 File — (Figure A) Aqueous humor sample step preparation for Hplc analysis. (Figure B) Graphical Abstract. (Table A) Ratio of phosphatidylcholine with Dorzolamide Hcl at low (A), medium (B) and high (C) pH values respectively. (Table B) Solubility Studies (Water/Octanol Solubility) of different SADN. (Table C) % Cumulative Release of Trusopt, SADN "F5 & F6". (DOCX) [file pone.0191415.s001.docx]

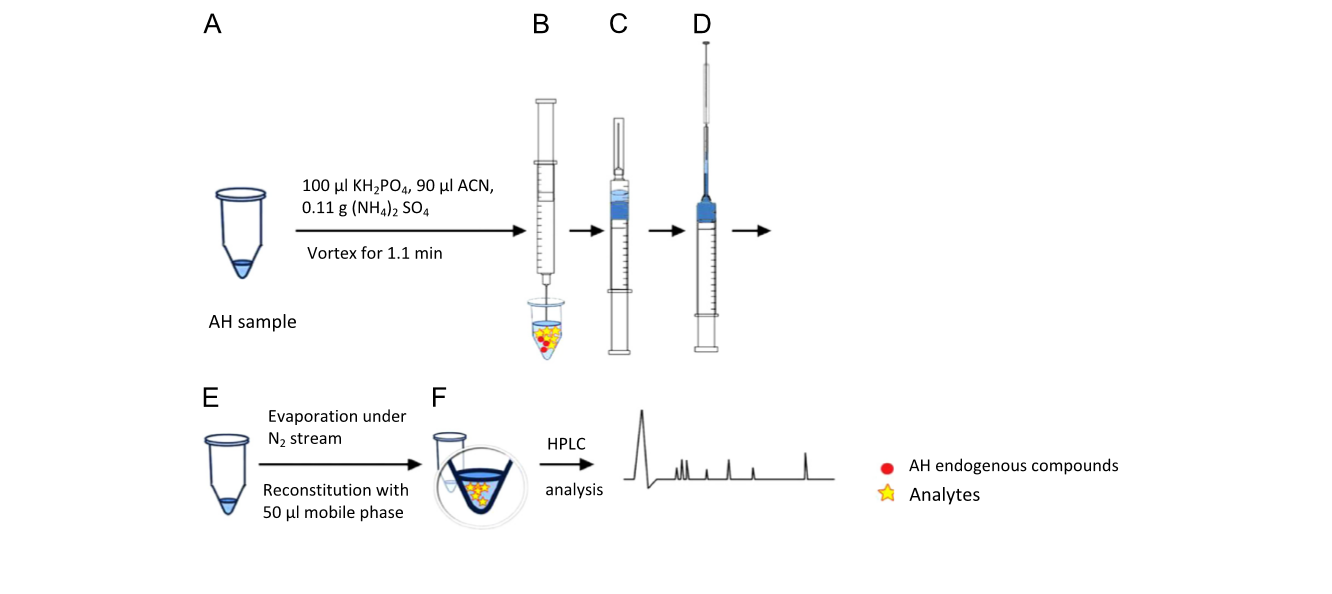
**Figure A: Aqueous humor sample step preparation for Hplc analysis**

**Figure B: Graphical Abstract:**


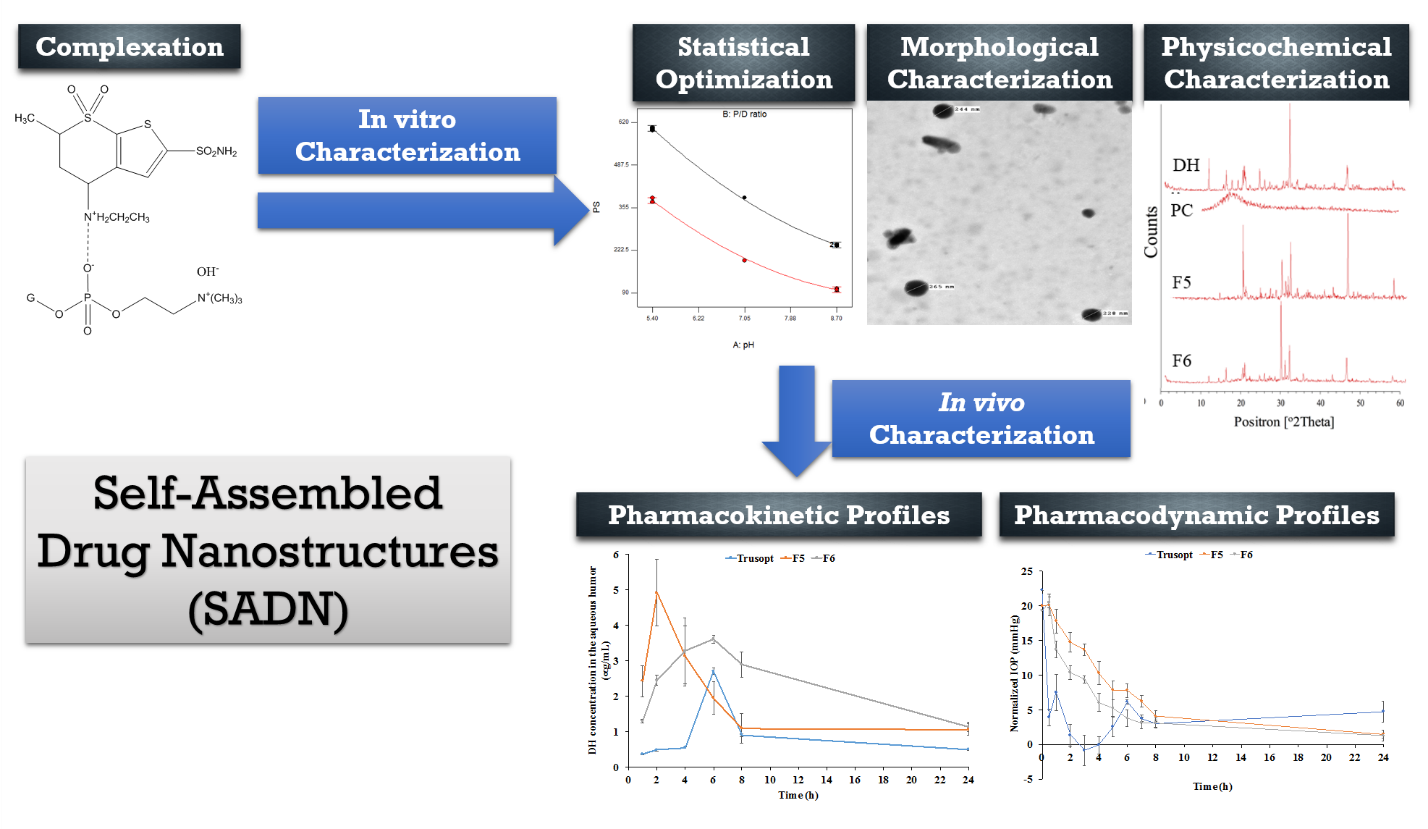


**Table A: Ratio of phosphatidylcholine with Dorzolamide Hcl at low (A), medium (B) and high (C) pH values** **respectively.**

| Formulae | pH | P/D ratio |
| --- | --- | --- |
| F1 | 5.4 | 1 |
|  |  |  |
| F2 | 5.4 | 2 |
|  |  |  |
| F3 | 7.05 | 1 |
| F4 | 7.05 | 2 |
| F5 | 8.7 | 1 |
|  |  |  |
| F6 | 8.7 | 2 |
|  |  |  |

**Table B: Solubility Studies (Water/Octanol Solubility) of different SADN.**

| **Formula No:** | **Constituent:** | **Solubility in Water %** | **Solubility in Octanol %** |
| --- | --- | --- | --- |
| **Fa** | **D.Hcl at pH 5.4** | **85.36** | **14.63** |
| **Fb** | **D.Hcl at pH 7.05** | **84.18** | **15.82** |
| **Fc** | **D.Hcl at pH 8.4** | **88.88** | **11.11** |
| **F1** | **SADN at pH 5.4 (1:1)** | **52.59** | **47.41** |
| **F2** | **SADN at pH 5.4 (1:2)** | **54.01** | **45.99** |
| **F3** | **SADN at pH 7.05 (1:1)** | **26.17** | **73.83** |
| **F4** | **SADN at pH 7.05 (1:2)** | **31.82** | **68.17** |
| **F5** | **SADN at pH 8.4 (1:1)** | **46.62** | **53.38** |
| **F6** | **SADN at pH 8.4 (1:2)** | **44.05** | **55.95** |

**Table C: % Cumulative Release of Trusopt, SADN "F5 & F6"**

| % Cumulative Release of Trusopt, SADN "F5 & F6" | | | |
| --- | --- | --- | --- |
| Time ( Hr) | Trusopt | SADN F5 | SADN F6 |
| 0 | 0.000 | 0 | 0 |
| 2 | 15.657 | 19.017 | 12.481 |
| 4 | 45.599 | 34.975 | 46.946 |
| 6 | 74.128 | 59.557 | 76.273 |
| 8 | 90.021 | 82.988 | 89.854 |
| 24 | ------- | 107.575 | 112.341 |
